# The Hidden Curriculum: A Good Thing?

**DOI:** 10.7759/cureus.6305

**Published:** 2019-12-06

**Authors:** Robin Mackin, Sue Baptiste, Anne Niec, April J Kam

**Affiliations:** 1 Pediatrics, McMaster Children's Hospital, Hamilton, CAN; 2 Rehabilitation Science, McMaster University, Hamilton, CAN; 3 Child Abuse, McMaster Children's Hospital, Hamilton, CAN; 4 Pediatric Emergency Medicine, Pediatrics, McMaster Children's Hospital, McMaster University, Hamilton, CAN

**Keywords:** medical education, professionalism, hidden curriculum

## Abstract

Introduction

The hidden curriculum is defined as a set of influences that function at the level of the organizational structure and culture to impact learning. Literature supports the significant impact of the hidden curriculum on all levels of learners in medical education. Our project aims to capture the messages being delivered to healthcare providers at our local facility.

Methods

Multiple one-time educational sessions on the hidden curriculum were provided over a five-year period to healthcare professionals. Participants were asked to share personal examples of their lived experiences with the hidden curriculum. A thematic analysis of the responses was completed and coded by two independent reviewers.

Results

Participants consisted of medical students, residents, faculty physicians, and allied health professionals. Their experience of the hidden curriculum emerged in six main themes: Vulnerability, Hierarchy, Privilege, Navigation & Negotiation, Positivity, and Dehumanizing.

Conclusion

A minority of responses demonstrated the positive impact that the hidden curriculum can have on professional development. This project highlights the importance of formally addressing the hidden curriculum to capitalize on its impact on medical trainees. The results have inspired a project focusing on residents as the population of interest in their unique role as learners and preceptors.

## Introduction

The hidden curriculum was first described by Dr. Frank Hafferty in the 1990s as a set of influences that function at the level of the organizational structure and culture to impact the learning environment [1]. He argues that most of what is learned in medical school is taught within medicine’s hidden curriculum. It contributes to a multidimensional learning environment that includes the formal, informal, and hidden curricula. The lessons communicated through the hidden curriculum extend beyond the classroom setting and formal educational objectives. It consists of messages communicated implicitly through everyday practices, habits, and vocabulary [2]. Every action performed or omitted, every joke, and every silence teach values that physicians might never have intended to impart [3]. The hidden curriculum is more than passive transmission of knowledge and skills but rather is a socialization process to the culture of medicine. The ‘classroom’ of the hidden curriculum takes place in corridors and call rooms with the potential to be more memorable than the explicit formal curriculum [3]. The hidden curriculum continues to gain attention by the medical education community due to the growing awareness of the significant impact on medical trainees’ professional development. In fact, the messages from the hidden curriculum often counter the formal curricular goals [4]. As described by Chuang et al, many students ‘face a professional identity conflict when the simple algorithms for patient interaction and disease management that were learned in the classroom fail to apply to the complex and dynamic clinical environment’ [5]. More so, the lessons from the hidden curriculum create a cognitive dissonance with trainees’ developed expectations distilled from the formal curricula [6].

The importance of the hidden curriculum has been organized into three main components as follows: it is critical to the professional and emotional growth of medical trainees; can benefit patient care as supported by the opportunity to develop more meaningful patient-physician relationships when a patient can trust their physician; and awareness and strategies generated to overcome the hidden curriculum may provide resilience to burn out and subsequently increase productivity of trainees [4].

Research on the hidden curriculum has shifted towards capturing and appreciating the lessons being transmitted to trainees. Gaufberg et al extracted nine core themes in the reflections of third-year medical students from Harvard Medical School [7]. Karnieli-Miller et al examined medical student narratives on professionalism for reference to components of the informal or hidden curriculum [8]. Other forms of qualitative research on the experienced hidden curriculum have included thematic analysis of focus groups with medical trainees [9]. To take control of the hidden curriculum, it is important to understand its presence and impact through the eyes of its audience. 

This phenomenology study attempts to capture the messages of the hidden curriculum by explicitly asking healthcare providers to share examples of their lived experiences. The aim of the study is to better understand the hidden curriculum at an academic tertiary care institution.

## Materials and methods

Over a five-year period (2013-2017), multiple (10) one-time educational sessions were provided to healthcare providers at McMaster University in Ontario, Canada. There were two consistent content expert facilitators for all of the sessions. The sessions included medical student lectures, grand rounds for Internal Medicine, Pediatrics, Ethics, and faculty development workshops. The predominance of workshops was delivered to medical students. The sessions were interactive through audience participation. Upon completion, participants could voluntarily share an example of the hidden curriculum from a personal experience by submitting a quote or short phrase on a blank cue card. Participant demographics were not collected in order to maintain anonymity. 

A total of 185 cue cards were submitted from the sessions. A thematic analysis was conducted by two independent reviewers over a three-month period. Manual coding of the data was completed by each individual and then reviewed in collaboration. This process was repeated three times before finalization of the themes. Each data point was organized and labeled under one of the six themes in order to appreciate the weighted distribution among the different themes. There were 18 cue cards that were not able to be classified under one of the six themes as a result of being hard to interpret or the writing was unable to be deciphered. 

Sessions on the hidden curriculum were offered as part of an already established curriculum for the medical school and health sciences faculty development; therefore, full ethics review was waived by the integrated research ethics board.

## Results

A total of 185 cue cards were collected from the 10 sessions over a five-year period at McMaster University with an interdisciplinary group representative of various departments, including but not limited to Internal Medicine, Pediatrics, and Surgery. The anonymous cue cards were written by staff physicians, residents, medical students, nurses, and other allied healthcare providers. A total of six themes were generated to represent the data: Vulnerability, Privilege, Dehumanizing , Hierarchy, Navigation & Negotiation, and Positivity. 

**Figure 1:**
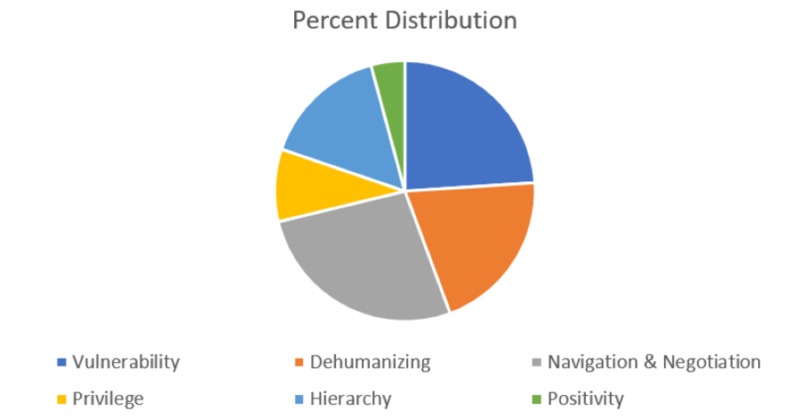
Percent distribution of comments among the six themes

As illustrated in Figure 1, 40 comments were classified under the theme of ‘Vulnerability’ (24%), 45 under the theme of ‘Navigation & Negotiation’ (26.9%), 34 under the theme of ‘Dehumanizing’ (20.4%), 26 under the theme ‘Hierarchy’ (15.6%), 15 under ‘Privilege’ (9%), and seven under the theme ‘Positivity’ (4.2%). Of the 185 cue cards, 18 comments were not included as a result of being unable to read the writing or interpret the content. 

**Figure 2:**
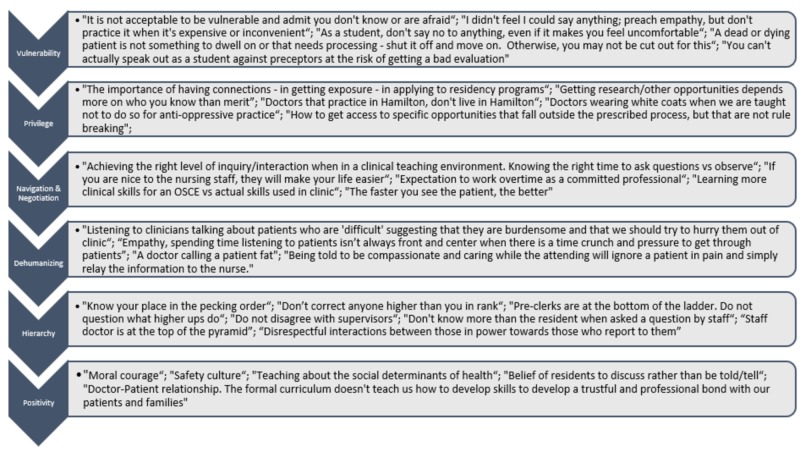
Examples of participants lived experiences with the hidden curriculum

A majority of the participants’ lived experiences with the hidden curriculum were related to professionalism, bias towards patients and trainees, and an expressed frustration with the disconnect between the way students are taught to practice medicine and what is observed in the clinical setting. Issues regarding ‘consent’ was one of the most recurring keywords in the data and often was classified under the theme ‘Dehumanizing’. Descriptions regarding the relationships between healthcare workers were commonly highlighted. More specifically, the nursing-physician relationship and the junior-senior medical trainee relationship were of particular interest. A few examples of the data obtained from our study have been included in Figure 2.

## Discussion

The data obtained support the strong presence of the hidden curriculum even in light of the fact that knowledge in this area of medicine has been available in academic journals since 1990 with ongoing lectures occurring at an academic tertiary care institution uncovering the issue for the past five years. The present study uncovered the six main themes of Vulnerability, Privilege, Navigation & Negotiation, Dehumanizing, Hierarchy, and Positivity. There was an overrepresentation of the themes ‘Vulnerability’ and ‘Navigation & Negotiation’ in this study. Given the predominance of medical student participants, it is not entirely surprising that early in their medical career they are more aware of and influenced by messages related to ‘surviving’ in the clinical environment. However, it is very concerning that content exposing the vulnerability of the healthcare provider is so strongly communicated. The predominant negative impact of the hidden curriculum is also highlighted by the fact that only 4.2% of the data could be classified under ‘Positivity’.

Although this study helps shed some light on the presence of the hidden curriculum at our center, the lack of participant demographics makes it hard to evaluate and fully understand the intricacies of its content. It would be interesting to know whether the distribution of themes would vary based on role in the healthcare system and level of training. Is it possible that people who have been in the clinical environment for longer periods of time become sensitized to the messages being conveyed through the hidden curriculum? Furthermore, are healthcare providers who have not received formal education on the hidden curriculum able to identify its presence in the work environment? This points to the importance of being aware of the hidden curriculum in order to truly understand its influence.

Compared to the study conducted by Gaufberg et al, where they generated a thematic analysis from the reflections of third-year medical students, both studies identified themes relating to ‘Hierarchy’, ‘Patient dehumanization’, and ‘Emotional Suppression/Vulnerability’ [7]. The concepts represented by our data can easily be applied to all nine of the themes identified in their study highlighting a consistency in the messaging that is being delivered through the hidden curriculum in medicine. The presence and impact of the hidden curriculum is not unique to our center. 

Dr. Robert Arnold described the term ‘cognitive dissonance’ as a disconnect between what is learned by a trainee in the pre-clinical years and what is experienced in the clinical environment [6]. Students are more inclined to consolidate learning by modeling what they have experienced or observed by their preceptors. In general, this is a very effective means of learning but a concern is raised when the actions being modeled counteract the intended learning goals and negatively impact a learners’ development. Evident in our data was the frustration experienced by participants when the expectations in the clinical environment were inconsistent with the messages relayed in pre-clinical years. For example, a quote from one participant, “unprofessional dialogue of staff physicians amongst themselves, while medical students are taught the value of appropriate interprofessional communication”. More concerning is the risk of medical trainees developing a sense of helplessness when faced with perceived unethical or unprofessional behavior. Another example from a participant, “we are taught to speak up against discrimination comments against patients/peers and to stop being bystanders - but how do we intervene, especially when we are not being directly addressed/included in that particular discussion? Is it my 'place' to bring up that issue with a superior”. The hidden curriculum can have a long lasting and detrimental impact on medical trainees if it is not addressed. Alternatively, its significant potential to positively influence medical trainees should become the focus of our efforts in taking control of the hidden curriculum.

Literature to date has focused on medical students as the area of interest in an attempt to better understand the messages being communicated. Residents provide a unique opportunity as a population to target given their concurrent role as learner and preceptor of junior trainees. By dedicating resources to help residents better understand and address the hidden curriculum, it can lead to sustainable change once they move into faculty positions with ongoing opportunities to be preceptors for trainees. As a definitive next step, the authors have developed new materials for a small group workshop for residents and fellows at their local institution with plans to study the effectiveness of the intervention on resident understanding of the hidden curriculum and their role in facilitating the hidden curriculum for junior trainees. Future research should also focus on trying to understand the challenges specific to faculty in order to impact culture and modeling behaviors. 

## Conclusions

There is no question that the hidden curriculum is present and impacting all domains of healthcare providers on a frequent basis. The themes captured in our data suggest that the hidden curriculum poses detrimental effects to trainees developing their professional identity and can directly impact patient care. It is extremely important to take control of the hidden curriculum and to capitalize on its potential to positively impact the professional development of our trainees. 
